# 2-{5-[2-(4-Nitro­phen­oxy)phen­yl]-1-phenyl-1*H*-pyrazol-3-yl}phenol

**DOI:** 10.1107/S1600536810008251

**Published:** 2010-03-10

**Authors:** Ali Haider, Zareen Akhter, Michael Bolte, Muhammad Zia-ul Haq, Humaira M. Siddiqi

**Affiliations:** a Department of Chemistry, Quaid-I-Azam University, Islamabad 45320, Pakistan; b Institut für Anorganische Chemie, J. W. Goethe-Universität Frankfurt, Max-von-Laue-Strasse 7, 60438 Frankfurt/Main, Germany; c National Engineering and Scientific Commission, PO Box 2216, Islamabad, Pakistan

## Abstract

In the title compound, C_27_H_19_N_3_O_4_, the phenol and pyrazole rings are almost coplanar [dihedral angle = 0.95 (12)°] due to an intra­molecular O—H⋯N hydrogen bond, whereas the phenyl ring is tilted by 40.81 (7)° with respect to the plane of the pyrazole ring. The aromatic ring with a nitro­phen­oxy substituent makes a dihedral angle of 54.10 (7)° with the pyrazole ring.

## Related literature

For pyrazole-containing derivatives, see: Habeeb *et al.* (2001 ▶); Hashimoto *et al.* (2002 ▶); Ranatunge *et al.* (2004 ▶); Elzein *et al.* (2006 ▶); Singh *et al.* (2005 ▶). For the properties and applications of aromatic polymers with diazole rings in the main chain, see: Bruma *et al.* (2003 ▶); Sava *et al.* (2003 ▶, 2006 ▶); Schulz *et al.* (1997 ▶). For the preparation of 2-(3-(2-hydroxy­phen­yl)-1-phenyl-1*H*-pyrazol-5-yl)phenol, see: Mukherjee (2000 ▶).
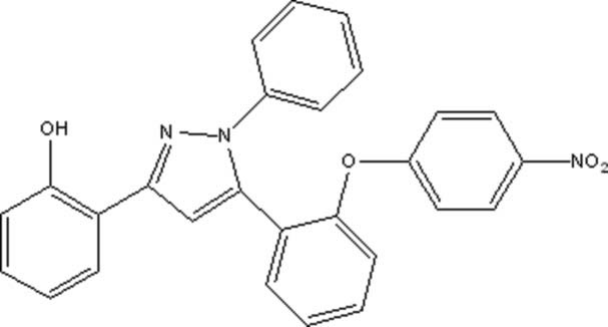


## Experimental

### 

#### Crystal data

C_27_H_19_N_3_O_4_
*M* *_r_* = 449.45
Monoclinic, 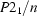

*a* = 12.1361 (12) Å
*b* = 10.9072 (12) Å
*c* = 16.6380 (16) Å
β = 98.081 (8)°
*V* = 2180.5 (4) Å^3^
*Z* = 4
Mo *K*α radiation
μ = 0.09 mm^−1^
*T* = 173 K
0.32 × 0.31 × 0.28 mm

#### Data collection

Stoe IPDS II two-circle diffractometer
12421 measured reflections
4064 independent reflections
2486 reflections with *I* > 2σ(*I*)
*R*_int_ = 0.057

#### Refinement

*R*[*F* ^2^ > 2σ(*F* ^2^)] = 0.037
*wR*(*F* ^2^) = 0.076
*S* = 0.81
4064 reflections
312 parameters
H atoms treated by a mixture of independent and constrained refinement
Δρ_max_ = 0.16 e Å^−3^
Δρ_min_ = −0.15 e Å^−3^

### 

Data collection: *X-AREA* (Stoe & Cie, 2001 ▶); cell refinement: *X-AREA*; data reduction: *X-AREA*; program(s) used to solve structure: *SHELXS97* (Sheldrick, 2008 ▶); program(s) used to refine structure: *SHELXL97* (Sheldrick, 2008 ▶); molecular graphics: *XP* (Sheldrick, 2008 ▶); software used to prepare material for publication: *SHELXL97*.

## Supplementary Material

Crystal structure: contains datablocks I, global. DOI: 10.1107/S1600536810008251/kp2252sup1.cif

Structure factors: contains datablocks I. DOI: 10.1107/S1600536810008251/kp2252Isup2.hkl

Additional supplementary materials:  crystallographic information; 3D view; checkCIF report

## Figures and Tables

**Table 1:** Hydrogen-bond geometry (Å, °)

*D*—H⋯*A*	*D*—H	H⋯*A*	*D*⋯*A*	*D*—H⋯*A*
O2—H2⋯N2	0.90 (2)	1.81 (3)	2.604 (2)	146 (2)

**Fig. 1.**
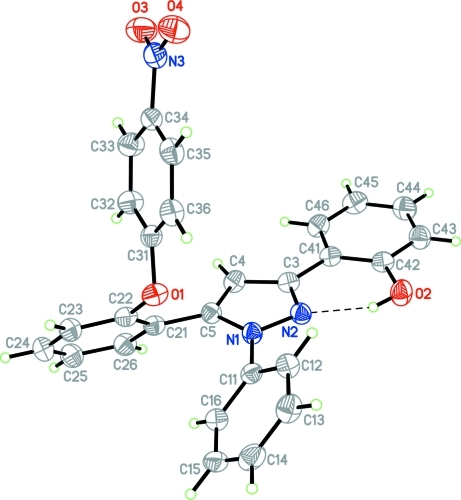
A view of the title compound with the atom-numbering scheme. Displacement ellipsoids are drawn at the 50% probability level and H atoms are shown as small spheres of arbitrary radii. The intramolecular hydrogen bond is shown as a dashed line.

## supplementary crystallographic information

### Comment

Pyrazole containing derivatives are attracted attention due to their biological properties and their outstanding functions as a part of aromatic polymer chains. The pyrazole unit is one of the core structures in a number of natural products and has been attracted attention in the field of biology (Habeeb *et al.*, 2001,  Hashimoto *et al.* 2002). Extensive studies have been devoted to arylpyrazole derivatives such as celecoxib,a well-known cyclooxygenase-2 inhibitor (Ranatunge *et al.*, 2004; Singh *et al.* 2005). Recently, pyrazole derivatives have been reported as high affinity and selective A2B adenosine receptor antagonist (Elzein *et al.*, 2006). On the other hand, it was shown that aromatic polymer with diazole rings in the main chain exhibit high thermal resistance in oxidative atmosphere, good hydrolytic stability, low dielectric permittivity, high toughness and other special properties which are determined by the electronic structure of this particular heterocycle (Schulz *et al.*, 1997; Sava *et al.*, 2003). The incorporation of oxadiazole and imide rings together with flexible groups into the polymer chain is expected to provide a combination of high-performance properties and processability (Bruma *et al.*, 2003, Sava *et al.*, 2006). The title compound, 2-(5-(2-(4-nitrophenoxy)phenyl)-1-phenyl-1*H*-pyrazol-3-yl)phenol has the prerequisite arylether linkages along with the hydroxyl and nitro-moieties and therefore can be an attractive synthon in material for biological application.

The *o*-phenol ring and the pyrazole ring in the title compound are almost coplanar [dihedral angle 0.95 (12)°] due to an intramolecular hydrogen bond, whereas the phenyl ring is tilted by 40.81 (7)° to the pyrazole ring. The aromatic ring carrying the nitrophenoxy substitutent makes a dihedral angle of 54.10 (7)° with the pyrazol ring. Crystal packing is determined by van der Waals interactions.

### Experimental

A mixture of 0.961 g (0.0061 mol) of 4- nitrophenol, 2 g (0.0061 mol) of synthesized 2-(3-(2-hydroxyphenyl)-1-phenyl-1*H*-pyrazol-5-yl)phenol (Mukherjee, 2000) and 0.842 g (0.0061 mol) of potassium carbonate in 50 ml of DMF was heated with stirring at 393 K for 12 h. The reaction mixture was cooled to room temperature and poured into 800 ml of ice cold water which resulted the yellow precipitation. After being washed repeatedly with water, the product was collected by filtration and was recrystallized from DMF to yield 72% of product (m.p. 474 K).

### Refinement

Hydrogen atoms bonded to C were included in calculated positions [C—H = 0.95 Å] and refined as riding [U_iso_(H) = 1.2U_eq_(C)]. The hydroxyl H atom was freely refined.

### Crystal data

**Table:**

C_27_H_19_N_3_O_4_	*F*(000) = 936
*M**_r_* = 449.45	*D*_x_ = 1.369 Mg m^−^^3^
Monoclinic, *P*2_1_/*n*	Mo *K*α radiation, λ = 0.71073 Å
Hall symbol: -P 2yn	Cell parameters from 6595 reflections
*a* = 12.1361 (12) Å	θ = 3.4–26.0°
*b* = 10.9072 (12) Å	µ = 0.09 mm^−^^1^
*c* = 16.6380 (16) Å	*T* = 173 K
β = 98.081 (8)°	Block, colourless
*V* = 2180.5 (4) Å^3^	0.32 × 0.31 × 0.28 mm
*Z* = 4	

### Data collection

**Table:**

Stoe IPDS II two-circle diffractometer	2486 reflections with *I* > 2σ(*I*)
Radiation source: fine-focus sealed tube	*R*_int_ = 0.057
graphite	θ_max_ = 25.6°, θ_min_ = 3.4°
ω scans	*h* = −14→14
12421 measured reflections	*k* = −11→13
4064 independent reflections	*l* = −20→18

### Refinement

**Table:**

Refinement on *F*^2^	Secondary atom site location: difference Fourier map
Least-squares matrix: full	Hydrogen site location: inferred from neighbouring sites
*R*[*F*^2^ > 2σ(*F*^2^)] = 0.037	H atoms treated by a mixture of independent and constrained refinement
*wR*(*F*^2^) = 0.076	*w* = 1/[σ^2^(*F*_o_^2^) + (0.0294*P*)^2^] where *P* = (*F*_o_^2^ + 2*F*_c_^2^)/3
*S* = 0.81	(Δ/σ)_max_ < 0.001
4064 reflections	Δρ_max_ = 0.16 e Å^−^^3^
312 parameters	Δρ_min_ = −0.15 e Å^−^^3^
0 restraints	Extinction correction: *SHELXL97* (Sheldrick, 2008), Fc^*^=kFc[1+0.001xFc^2^λ^3^/sin(2θ)]^-1/4^
Primary atom site location: structure-invariant direct methods	Extinction coefficient: 0.0067 (5)

### Special details

**Table:**

Geometry. All esds (except the esd in the dihedral angle between two l.s. planes) are estimated using the full covariance matrix. The cell esds are taken into account individually in the estimation of esds in distances, angles and torsion angles; correlations between esds in cell parameters are only used when they are defined by crystal symmetry. An approximate (isotropic) treatment of cell esds is used for estimating esds involving l.s. planes.
Refinement. Refinement of *F*^2^ against ALL reflections. The weighted *R*-factor wR and goodness of fit *S* are based on *F*^2^, conventional *R*-factors *R* are based on *F*, with *F* set to zero for negative *F*^2^. The threshold expression of *F*^2^ > σ(*F*^2^) is used only for calculating *R*-factors(gt) etc. and is not relevant to the choice of reflections for refinement. *R*-factors based on *F*^2^ are statistically about twice as large as those based on *F*, and *R*- factors based on ALL data will be even larger.

### Fractional atomic coordinates and isotropic or equivalent isotropic displacement parameters (Å^2^)

**Table:**

	*x*	*y*	*z*	*U*_iso_*/*U*_eq_	
N1	0.65535 (10)	0.61876 (14)	0.45161 (9)	0.0274 (3)	
N2	0.57870 (10)	0.70777 (14)	0.42624 (9)	0.0286 (4)	
N3	1.03923 (12)	1.04191 (16)	0.68913 (12)	0.0408 (4)	
C3	0.53351 (12)	0.73920 (17)	0.49247 (11)	0.0278 (4)	
C4	0.58124 (12)	0.67048 (18)	0.56011 (11)	0.0306 (4)	
H4	0.5634	0.6751	0.6138	0.037*	
C5	0.65914 (12)	0.59517 (17)	0.53256 (11)	0.0273 (4)	
O1	0.89602 (9)	0.60627 (12)	0.53359 (7)	0.0312 (3)	
O2	0.44973 (10)	0.86574 (14)	0.34163 (8)	0.0376 (3)	
H2	0.5028 (18)	0.808 (2)	0.3516 (15)	0.067 (8)*	
O3	1.00823 (12)	1.06753 (14)	0.75453 (10)	0.0531 (4)	
O4	1.09471 (11)	1.11286 (14)	0.65330 (10)	0.0533 (4)	
C11	0.71384 (12)	0.56211 (17)	0.39239 (11)	0.0272 (4)	
C12	0.75161 (13)	0.63578 (18)	0.33421 (11)	0.0315 (4)	
H12	0.7398	0.7219	0.3341	0.038*	
C13	0.80707 (13)	0.5813 (2)	0.27611 (12)	0.0378 (5)	
H13	0.8323	0.6304	0.2352	0.045*	
C14	0.82606 (14)	0.4566 (2)	0.27703 (12)	0.0408 (5)	
H14	0.8650	0.4205	0.2373	0.049*	
C15	0.78832 (14)	0.3838 (2)	0.33594 (12)	0.0382 (5)	
H15	0.8018	0.2980	0.3367	0.046*	
C16	0.73091 (13)	0.43615 (18)	0.39379 (12)	0.0326 (4)	
H16	0.7037	0.3866	0.4337	0.039*	
C21	0.73660 (12)	0.50662 (17)	0.57734 (11)	0.0280 (4)	
C22	0.85183 (13)	0.51311 (17)	0.57759 (11)	0.0277 (4)	
C23	0.92304 (14)	0.42891 (18)	0.61848 (12)	0.0341 (5)	
H23	1.0006	0.4333	0.6156	0.041*	
C24	0.88194 (15)	0.33786 (19)	0.66389 (13)	0.0392 (5)	
H24	0.9311	0.2803	0.6930	0.047*	
C25	0.76912 (15)	0.33115 (19)	0.66670 (13)	0.0388 (5)	
H25	0.7406	0.2693	0.6983	0.047*	
C26	0.69702 (14)	0.41432 (18)	0.62353 (12)	0.0335 (5)	
H26	0.6194	0.4082	0.6255	0.040*	
C31	0.93323 (12)	0.70939 (17)	0.57673 (11)	0.0274 (4)	
C32	0.91071 (14)	0.73406 (18)	0.65426 (12)	0.0341 (5)	
H32	0.8705	0.6767	0.6818	0.041*	
C33	0.94728 (14)	0.84307 (19)	0.69138 (12)	0.0382 (5)	
H33	0.9312	0.8618	0.7443	0.046*	
C34	1.00691 (13)	0.92394 (17)	0.65128 (12)	0.0318 (4)	
C35	1.03368 (13)	0.89819 (18)	0.57519 (12)	0.0335 (5)	
H35	1.0774	0.9539	0.5492	0.040*	
C36	0.99614 (13)	0.79031 (18)	0.53732 (12)	0.0317 (4)	
H36	1.0132	0.7715	0.4847	0.038*	
C41	0.44668 (12)	0.83512 (17)	0.48557 (11)	0.0284 (4)	
C42	0.40952 (13)	0.89354 (18)	0.41212 (11)	0.0304 (4)	
C43	0.32725 (13)	0.98405 (19)	0.40739 (12)	0.0367 (5)	
H43	0.3022	1.0227	0.3569	0.044*	
C44	0.28269 (14)	1.01707 (19)	0.47573 (13)	0.0396 (5)	
H44	0.2267	1.0785	0.4724	0.047*	
C45	0.31869 (13)	0.96169 (19)	0.54894 (13)	0.0384 (5)	
H45	0.2881	0.9855	0.5961	0.046*	
C46	0.39962 (13)	0.87122 (18)	0.55412 (12)	0.0347 (5)	
H46	0.4235	0.8331	0.6049	0.042*	

### Atomic displacement parameters (Å^2^)

**Table:**

	*U*^11^	*U*^22^	*U*^33^	*U*^12^	*U*^13^	*U*^23^
N1	0.0278 (7)	0.0289 (9)	0.0260 (9)	0.0040 (6)	0.0051 (6)	0.0014 (7)
N2	0.0287 (7)	0.0283 (9)	0.0292 (9)	0.0042 (6)	0.0050 (6)	−0.0002 (7)
N3	0.0400 (8)	0.0317 (10)	0.0474 (12)	−0.0007 (7)	−0.0058 (8)	0.0061 (9)
C3	0.0255 (8)	0.0306 (11)	0.0285 (11)	−0.0022 (7)	0.0074 (7)	−0.0023 (9)
C4	0.0299 (8)	0.0367 (12)	0.0262 (10)	−0.0001 (8)	0.0078 (7)	−0.0001 (9)
C5	0.0284 (8)	0.0276 (11)	0.0261 (10)	−0.0017 (7)	0.0051 (7)	0.0025 (8)
O1	0.0335 (6)	0.0338 (8)	0.0273 (7)	−0.0074 (5)	0.0080 (5)	−0.0027 (6)
O2	0.0424 (7)	0.0438 (9)	0.0278 (8)	0.0089 (6)	0.0094 (6)	0.0037 (7)
O3	0.0711 (9)	0.0403 (10)	0.0465 (10)	−0.0033 (7)	0.0033 (8)	−0.0080 (8)
O4	0.0550 (8)	0.0366 (9)	0.0659 (11)	−0.0147 (7)	0.0001 (7)	0.0073 (9)
C11	0.0250 (8)	0.0314 (11)	0.0249 (10)	0.0014 (7)	0.0027 (7)	−0.0039 (9)
C12	0.0312 (8)	0.0327 (11)	0.0312 (11)	0.0015 (8)	0.0067 (7)	0.0015 (10)
C13	0.0346 (9)	0.0493 (14)	0.0304 (12)	0.0051 (9)	0.0084 (8)	0.0015 (10)
C14	0.0342 (9)	0.0555 (15)	0.0319 (12)	0.0109 (9)	0.0024 (8)	−0.0108 (11)
C15	0.0401 (9)	0.0345 (12)	0.0375 (12)	0.0094 (8)	−0.0029 (8)	−0.0119 (11)
C16	0.0350 (9)	0.0294 (11)	0.0321 (11)	−0.0003 (8)	0.0007 (8)	−0.0008 (9)
C21	0.0307 (8)	0.0279 (11)	0.0257 (10)	−0.0016 (7)	0.0045 (7)	−0.0026 (9)
C22	0.0314 (8)	0.0260 (11)	0.0261 (10)	−0.0037 (7)	0.0056 (7)	−0.0031 (9)
C23	0.0328 (9)	0.0341 (12)	0.0347 (11)	0.0042 (8)	0.0017 (8)	−0.0027 (10)
C24	0.0428 (10)	0.0316 (12)	0.0409 (12)	0.0069 (9)	−0.0019 (8)	0.0017 (10)
C25	0.0495 (11)	0.0294 (11)	0.0369 (12)	−0.0049 (9)	0.0043 (9)	0.0077 (10)
C26	0.0340 (9)	0.0324 (12)	0.0343 (11)	−0.0050 (8)	0.0055 (8)	0.0017 (10)
C31	0.0238 (8)	0.0289 (11)	0.0292 (11)	0.0013 (7)	0.0022 (7)	−0.0009 (9)
C32	0.0402 (9)	0.0333 (12)	0.0304 (11)	−0.0071 (8)	0.0101 (8)	0.0012 (10)
C33	0.0492 (10)	0.0362 (12)	0.0300 (11)	−0.0045 (9)	0.0088 (9)	−0.0014 (10)
C34	0.0315 (8)	0.0259 (11)	0.0355 (11)	−0.0016 (7)	−0.0035 (8)	0.0025 (9)
C35	0.0280 (8)	0.0317 (12)	0.0414 (12)	−0.0018 (8)	0.0067 (8)	0.0081 (10)
C36	0.0303 (9)	0.0349 (12)	0.0312 (11)	0.0010 (8)	0.0093 (8)	0.0051 (10)
C41	0.0260 (8)	0.0300 (11)	0.0298 (11)	−0.0007 (7)	0.0066 (7)	−0.0027 (9)
C42	0.0293 (8)	0.0317 (11)	0.0311 (11)	−0.0014 (8)	0.0070 (7)	−0.0029 (9)
C43	0.0338 (9)	0.0385 (13)	0.0376 (12)	0.0047 (8)	0.0047 (8)	0.0024 (10)
C44	0.0279 (8)	0.0406 (13)	0.0502 (14)	0.0061 (8)	0.0052 (8)	−0.0034 (11)
C45	0.0305 (9)	0.0474 (14)	0.0394 (12)	0.0030 (8)	0.0119 (8)	−0.0093 (11)
C46	0.0317 (8)	0.0428 (13)	0.0302 (11)	0.0009 (8)	0.0064 (7)	−0.0016 (10)

### Geometric parameters (Å, °)

**Table:**

N1—C5	1.366 (2)	C22—C23	1.374 (3)
N1—N2	1.370 (2)	C23—C24	1.383 (3)
N1—C11	1.433 (2)	C23—H23	0.9500
N2—C3	1.343 (2)	C24—C25	1.378 (3)
N3—O3	1.232 (2)	C24—H24	0.9500
N3—O4	1.233 (2)	C25—C26	1.388 (3)
N3—C34	1.462 (3)	C25—H25	0.9500
C3—C4	1.407 (3)	C26—H26	0.9500
C3—C41	1.478 (2)	C31—C32	1.382 (3)
C4—C5	1.379 (2)	C31—C36	1.390 (2)
C4—H4	0.9500	C32—C33	1.384 (3)
C5—C21	1.475 (2)	C32—H32	0.9500
O1—C31	1.376 (2)	C33—C34	1.372 (3)
O1—C22	1.402 (2)	C33—H33	0.9500
O2—C42	1.366 (2)	C34—C35	1.379 (3)
O2—H2	0.90 (2)	C35—C36	1.381 (3)
C11—C12	1.385 (3)	C35—H35	0.9500
C11—C16	1.389 (3)	C36—H36	0.9500
C12—C13	1.387 (3)	C41—C42	1.396 (3)
C12—H12	0.9500	C41—C46	1.402 (2)
C13—C14	1.380 (3)	C42—C43	1.398 (3)
C13—H13	0.9500	C43—C44	1.374 (3)
C14—C15	1.388 (3)	C43—H43	0.9500
C14—H14	0.9500	C44—C45	1.375 (3)
C15—C16	1.388 (3)	C44—H44	0.9500
C15—H15	0.9500	C45—C46	1.386 (3)
C16—H16	0.9500	C45—H45	0.9500
C21—C26	1.392 (3)	C46—H46	0.9500
C21—C22	1.400 (2)		
			
C5—N1—N2	111.39 (14)	C25—C24—C23	119.53 (18)
C5—N1—C11	130.22 (15)	C25—C24—H24	120.2
N2—N1—C11	118.32 (14)	C23—C24—H24	120.2
C3—N2—N1	105.44 (15)	C24—C25—C26	120.30 (19)
O3—N3—O4	122.67 (18)	C24—C25—H25	119.9
O3—N3—C34	118.89 (17)	C26—C25—H25	119.9
O4—N3—C34	118.42 (18)	C25—C26—C21	121.11 (16)
N2—C3—C4	110.53 (15)	C25—C26—H26	119.4
N2—C3—C41	119.08 (17)	C21—C26—H26	119.4
C4—C3—C41	130.39 (16)	O1—C31—C32	123.54 (16)
C5—C4—C3	106.01 (15)	O1—C31—C36	115.77 (16)
C5—C4—H4	127.0	C32—C31—C36	120.69 (17)
C3—C4—H4	127.0	C31—C32—C33	119.39 (18)
N1—C5—C4	106.63 (15)	C31—C32—H32	120.3
N1—C5—C21	123.53 (15)	C33—C32—H32	120.3
C4—C5—C21	129.83 (16)	C34—C33—C32	119.56 (18)
C31—O1—C22	116.21 (13)	C34—C33—H33	120.2
C42—O2—H2	109.2 (16)	C32—C33—H33	120.2
C12—C11—C16	121.49 (17)	C33—C34—C35	121.58 (18)
C12—C11—N1	118.36 (16)	C33—C34—N3	118.97 (18)
C16—C11—N1	120.14 (17)	C35—C34—N3	119.41 (17)
C11—C12—C13	118.65 (18)	C34—C35—C36	119.14 (17)
C11—C12—H12	120.7	C34—C35—H35	120.4
C13—C12—H12	120.7	C36—C35—H35	120.4
C14—C13—C12	120.76 (19)	C35—C36—C31	119.57 (17)
C14—C13—H13	119.6	C35—C36—H36	120.2
C12—C13—H13	119.6	C31—C36—H36	120.2
C13—C14—C15	120.03 (18)	C42—C41—C46	117.76 (16)
C13—C14—H14	120.0	C42—C41—C3	122.02 (16)
C15—C14—H14	120.0	C46—C41—C3	120.22 (17)
C16—C15—C14	120.2 (2)	O2—C42—C41	122.68 (16)
C16—C15—H15	119.9	O2—C42—C43	116.53 (17)
C14—C15—H15	119.9	C41—C42—C43	120.79 (17)
C15—C16—C11	118.91 (19)	C44—C43—C42	119.95 (19)
C15—C16—H16	120.5	C44—C43—H43	120.0
C11—C16—H16	120.5	C42—C43—H43	120.0
C26—C21—C22	117.19 (16)	C43—C44—C45	120.34 (18)
C26—C21—C5	120.50 (14)	C43—C44—H44	119.8
C22—C21—C5	122.28 (16)	C45—C44—H44	119.8
C23—C22—C21	121.75 (17)	C44—C45—C46	120.14 (18)
C23—C22—O1	118.87 (14)	C44—C45—H45	119.9
C21—C22—O1	119.37 (15)	C46—C45—H45	119.9
C22—C23—C24	120.04 (16)	C45—C46—C41	121.02 (18)
C22—C23—H23	120.0	C45—C46—H46	119.5
C24—C23—H23	120.0	C41—C46—H46	119.5
			
C5—N1—N2—C3	−0.50 (19)	C22—C23—C24—C25	1.0 (3)
C11—N1—N2—C3	176.84 (14)	C23—C24—C25—C26	0.7 (3)
N1—N2—C3—C4	0.04 (19)	C24—C25—C26—C21	−0.6 (3)
N1—N2—C3—C41	179.75 (15)	C22—C21—C26—C25	−1.1 (3)
N2—C3—C4—C5	0.4 (2)	C5—C21—C26—C25	−179.30 (18)
C41—C3—C4—C5	−179.25 (18)	C22—O1—C31—C32	−12.3 (2)
N2—N1—C5—C4	0.76 (19)	C22—O1—C31—C36	167.88 (15)
C11—N1—C5—C4	−176.16 (16)	O1—C31—C32—C33	−176.95 (16)
N2—N1—C5—C21	−177.86 (15)	C36—C31—C32—C33	2.8 (3)
C11—N1—C5—C21	5.2 (3)	C31—C32—C33—C34	−1.1 (3)
C3—C4—C5—N1	−0.69 (19)	C32—C33—C34—C35	−1.5 (3)
C3—C4—C5—C21	177.80 (17)	C32—C33—C34—N3	176.35 (16)
C5—N1—C11—C12	−141.18 (18)	O3—N3—C34—C33	−2.9 (2)
N2—N1—C11—C12	42.1 (2)	O4—N3—C34—C33	178.57 (17)
C5—N1—C11—C16	39.2 (3)	O3—N3—C34—C35	174.99 (17)
N2—N1—C11—C16	−137.50 (16)	O4—N3—C34—C35	−3.6 (2)
C16—C11—C12—C13	0.4 (3)	C33—C34—C35—C36	2.3 (3)
N1—C11—C12—C13	−179.21 (15)	N3—C34—C35—C36	−175.46 (15)
C11—C12—C13—C14	−1.2 (3)	C34—C35—C36—C31	−0.6 (2)
C12—C13—C14—C15	0.8 (3)	O1—C31—C36—C35	177.84 (14)
C13—C14—C15—C16	0.3 (3)	C32—C31—C36—C35	−1.9 (3)
C14—C15—C16—C11	−1.1 (3)	N2—C3—C41—C42	0.5 (3)
C12—C11—C16—C15	0.8 (3)	C4—C3—C41—C42	−179.89 (18)
N1—C11—C16—C15	−179.68 (15)	N2—C3—C41—C46	−178.83 (16)
N1—C5—C21—C26	−127.17 (19)	C4—C3—C41—C46	0.8 (3)
C4—C5—C21—C26	54.6 (3)	C46—C41—C42—O2	179.91 (16)
N1—C5—C21—C22	54.8 (3)	C3—C41—C42—O2	0.6 (3)
C4—C5—C21—C22	−123.5 (2)	C46—C41—C42—C43	−0.7 (3)
C26—C21—C22—C23	2.9 (3)	C3—C41—C42—C43	−179.97 (17)
C5—C21—C22—C23	−178.94 (18)	O2—C42—C43—C44	180.00 (17)
C26—C21—C22—O1	−178.47 (16)	C41—C42—C43—C44	0.5 (3)
C5—C21—C22—O1	−0.4 (3)	C42—C43—C44—C45	0.1 (3)
C31—O1—C22—C23	−85.43 (19)	C43—C44—C45—C46	−0.6 (3)
C31—O1—C22—C21	95.94 (18)	C44—C45—C46—C41	0.5 (3)
C21—C22—C23—C24	−2.9 (3)	C42—C41—C46—C45	0.2 (3)
O1—C22—C23—C24	178.47 (17)	C3—C41—C46—C45	179.50 (18)

### Hydrogen-bond geometry (Å, °)

**Table:**

*D*—H···*A*	*D*—H	H···*A*	*D*···*A*	*D*—H···*A*
O2—H2···N2	0.90 (2)	1.81 (3)	2.604 (2)	146 (2)
